# Investigation of an outbreak of mycobacteriosis in pigs

**DOI:** 10.1186/1746-6148-7-63

**Published:** 2011-10-21

**Authors:** Angelika Agdestein, Tone B Johansen, Vladimir Polaček, Bjørn Lium, Gudmund Holstad, Dejan Vidanović, Sanja Aleksić-Kovačević, Anne Jørgensen, Jonas Žultauskas, Sigrun F Nilsen, Berit Djønne

**Affiliations:** 1 Norwegian Veterinary Institute, P. O. Box 750 dep., N-0106 Oslo, Norway; 2 Veterinary Specialized Institute "Kraljevo", Žička 34, 36000 Kraljevo, Serbia; 3 Norwegian School of Veterinary Science, P. O. Box 8156 Dep., N-0033 Oslo, Norway; 4 Department of Pathology, Faculty of Veterinary Medicine, Belgrade University, Bulevar Oslobođenja 18, 11000 Belgrade, Serbia; 5 Norwegian Pig Health Service, Animalia, P.O. Box 396 Økern, N-0513 OSLO, Norway; 6 Norsvin Lithuania, Mūro Strèvininkai, 56202 Kaišiadoriųraj, Lithuania

## Abstract

**Background:**

A high proportion of pigs imported to Serbia from a Lithuanian breeding herd reacted positively against avian and/or bovine tuberculin. The pigs were euthanized and lesions characteristic for mycobacterial infection were detected. An investigation of potential mycobacteriosis in the pigs imported to Serbia and the possible source of infection in the Lithuanian herd were therefore initialised.

**Results:**

Formalin fixed, paraffin embedded lymph nodes from tuberculin positive animals were examined by real-time PCR for IS*1245 *and IS*6110*. IS*1245 *was detected in 55% and IS*6110 *in 11% of the samples. Seven of the ten IS*6110 *positive samples were positive for IS*1245*. Eleven lymph nodes from 10 pigs and 15 environmental samples were collected from the Lithuanian breeding herd and cultured for mycobacteria. *M. avium *subsp. *hominissuis *was detected in all lymph nodes and from eight samples of peat and sawdust. Isolates with identical and related IS*1245*- and IS*1311 *RFLP profiles were detected from swine and peat.

**Conclusions:**

This study demonstrated cross reactions between avian and bovine tuberculin in pigs. Real-time PCR indicated infection with *M. avium *in the Serbian pigs. However, as a small proportion of the lymph nodes were positive for IS*6110*, infection with bacteria in the *M. tuberculosis *complex could not be ruled out. Analyses confirmed the presence of *M. avium *subsp. *hominissuis *in porcine and environmental samples from the Lithuanian breeding herd. The results indicate peat as a source of *M. avium *subsp. *hominissuis *infection in these pigs, and that the pigs imported to Serbia were infected with *M. avium *subsp. *hominissuis*.

## Background

*Mycobacterium avium*, belonging to the *Mycobacterium avium *complex (MAC), has been divided into the subspecies *avium*, *paratuberculosis *and *silvaticum *[1]. More recently, *M. avium *subsp. *avium *has been further divided into *M. avium *subsp. *avium *and *M. avium *subsp. *hominissuis *[2]. *M. avium *has been isolated from different sources, such as water, pools, soil, plants and bedding material [3]. *M. avium *subsp. *avium *mainly cause generalised tuberculosis in poultry and wild birds, while *M. avium *subsp. *hominissuis *is an opportunistic pathogen, infecting mainly swine and humans [2,4,5]. *M. bovis *is the main agent causing tuberculosis in cattle, while *M. tuberculosis *primarily causes tuberculosis in humans. Both belong to the *M. tuberculosis *complex (MTC) and can lead to infections in pigs. Mycobacteriosis in pigs is mainly caused by *M. avium *subsp. *hominissuis*, and infection can lead to economic losses due to condemnation of carcasses. However, the MTC can cause similar lesions, and is a differential diagnosis with serious implications for the herd [6-8].

Intradermal tuberculin testing can be used for ante mortem diagnosis of tuberculosis in pigs [4,9]. The single intradermal comparative test entails simultaneous injection of bovine and avian tuberculin. Animals infected with MTC tend to show greater reaction to bovine tuberculin than to avian tuberculin, while animals exposed for *M. avium *or other mycobacteria tend to promote the reverse reaction. Cross reactions are seen, but although the sensitivity and the specificity of the test are imperfect, the test has an important function, especially in herd diagnosis [9,10]. Post mortem, mycobacterial infections can be diagnosed by pathological examination combined with histopathology. The histological diagnosis of tuberculosis comprises detection and localisation of acid fast bacilli in granulomatous lesions [11]. Culturing of mycobacteria is the gold standard for diagnosis, but molecular tests are also used [4,12]. Formalin fixed tissue can be used for analysis by PCR, and real time PCR is considered more sensitive than conventional PCR for such material [13]. Different genetic elements as the mobile insertion sequences (IS) can be used for diagnosis and genetic typing of mycobacteria. IS*1245 *is considered specific for *M. avium *[14] and IS*6110 *for the MTC [15], whereas IS*901 *is found only in *M. avium *subsp. *avium *[16].

In June 2007, 217 pigs were exported from a breeding herd in Lithuania to Serbia. All pigs were born between December 2006 and April 2007. During the quarantine period in Serbia, the pigs were skin tested with both avian and bovine tuberculin, as ordered by the Serbian Veterinary Directorate. Altogether 62 pigs reacted positively against avian and/or bovine tuberculin. One pig was euthanized, and lesions compatible with mycobacteriosis were revealed. According to instructions from the Serbian Veterinary Directorate, all tuberculin positive pigs were euthanized. The aim of the present study was to examine the remaining pigs imported to Serbia in order to determine whether they were infected by mycobacteria able to cause lesions and tuberculin reactions as observed. In addition, the possible source of infection in the Lithuanian breeding herd was investigated.

## Methods

### Tuberculin testing and post mortem examinations of pigs imported to Serbia

Tuberculin retesting of 154 out of the 155 pigs negative on the first examination was performed two months after the first test, a time interval regarded as sufficient to allow desensitation to wane [12]. Both avian and bovine tuberculin were used as indicated by the producer (Bioveta, Ivanovicena Hané, Czech Republic), followed by rigorous interpretation of results [12]. Thereafter, all remaining pigs were euthanized and examined for lesions compatible with tuberculosis, such as enlargement, miliar necrosis, caseous necrosis and calcification. Formalin fixation and paraffin embedding (FFPE) of lymph nodes from tuberculin positive pigs was performed as described [17]. The paraffin sections (3-5 μm thick) were stained with haematoxylin and eosin (HE) and Ziehl-Neelsen (ZN) staining for microscopic examination and detection of acid fast bacteria.

Samples from 25 randomly chosen tuberculin positive pigs with gross lesions characteristic for mycobacteriosis were sent for culturing of mycobacteria at the Department of Tuberculosis, Clinical Centre in Kraljevo, Serbia. Approximately 2 grams of tissue from mesenterial lymph nodes were grinded and decontaminated with 2% NaOH. Isolation was performed on Löwenstein-Jensen medium with glycerol at 37°C for 8 weeks [12].

### Real-time PCR from FFPE lymph nodes from pigs imported to Serbia

One hundred and fifteen FFPE lymph nodes from 100 pigs were sent to the Norwegian Veterinary Institute, and real-time PCRs for detection of DNA sequences of *M. avium *and MTC were performed. For extraction of DNA Nucleon HT (Tepnel Life Sciences, Manchester, UK) was applied, following the protocol provided by the manufacturer. From mycobacterial isolates and non-fixed tissue samples, DNA was extracted by Nuclisens^® ^easyMag^® ^(BioMérieux, Inc., Durham, NC, USA) following the manufacturer's instructions. Three singleplex real-time PCRs were developed, for IS*1245 *and IS*6110 *specific for *M. avium *and MTC respectively, and for the porcine β-globin gene. Amplification of the latter sequence was used as a positive control for successful DNA extraction from the samples. Primers and 6FAM labelled TaqMan MGB probes were designed using the program Primer 3.0 http://frodo.wi.mit.edu/primer3/ (Table 1). Real-time PCR was performed using Stratagene Mx3005P (Stratagene, La Jolla, CA, USA). Reaction mixtures had a total volume of 20 μl, consisting of 2 μl template DNA at a concentration of 10-15 ng/μl, 10 μl PerfeC Ta^® ^qPCR FastMix^® ^(Quanta Biosciences, Gaithersburg, MD, USA), and primers and probes at a final concentration of 400 and 150 nM, respectively.

**Table 1:** Primers and probes used in this study

Target	Size of amplified sequence (bp)	Primers and probes	**GenBank accession no**.
IS*1245*	82	Primer 41: ggtgagcggatcactcaag* Primer 116: ggagaagccccgatgaac Probe 3: caagccttgatcgacgcgga	L33879

IS*6110*	101	Primer 149: gccaactacggtgtttacgg Primer 150: agtttggtcatcagccgttc Probe 6: gggcatcgaggtggccagat	X17348.1

Porcine β-globin gene	119	Primer 120: gggggttgcaatttattcct Primer 121: tgaatcacggtcctgtgaaa Probe 4: cgcagattcccaaaccttcgc	X86791

*[18]

DNA extracted from FFPE lymph nodes from animals clinically infected with *M. avium *subsp. *hominissuis *or *M. bovis*, verified by culture prior to fixation, were used for optimisation of PCR conditions. DNA extracted from *M. avium *ATCC 25291, *M. bovis *BCG (Danish strain 1331) and from a porcine spleen, were used as positive controls for IS*1245*, IS*6110 *and β-globin. Ultrapure Milli-Q water was used for adjustment of volume and concentrations and as a template substitute in negative controls.

The real-time PCR reactions were initiated with denaturation at 95°C for 10 min, followed by 45 runs of the following thermal cycle: 95°C for 3 sec and 60°C for 30 sec. All samples and controls were run as single measurements. The results were analysed using Stratagene MxPro 4.10 software (Stratagene), applying the automatic calculation of the threshold fluorescence. Ct values above 40 were regarded as negative as recommended by the manufacturer (Stratagene).

### Post mortem examinations of animals and examinations of environmental samples from the Lithuanian herd

Six cervical and five mesenterial lymph nodes from ten randomly selected, clinically healthy pigs from the original Lithuanian herd were collected at slaughter for gross pathology, culturing and typing of mycobacteria. Gross pathology of the lymph nodes was evaluated upon incision. Thereafter, culturing of the samples was performed as described previously [18,19]. Briefly, approximately two grams of tissue from lymph nodes or internal organs were homogenised, decontaminated with 5% oxalic acid and inoculated on different media with and without pyruvate and incubated at 37°C for up to two months [18].

Samples from the herd facilities; four from tap water, six from peat, four from sawdust and one scraping from the inside of a water pipe were analysed. The samples of peat and sawdust were taken from storage facilities, and had not been in contact with the pigs before sampling. All samples are described in Table 2. Pre-treatment of the environmental samples was performed slightly different depending on the type of material. Samples from peat and sawdust were soaked in an equal amount of sterile distilled water for 24 h. Water samples were centrifuged at 3500 rpm for 15 minutes and the pellets were processed further. For all environmental samples, decontamination was performed by 4% NaOH followed by 5% oxalic acid with 0.1% malachite green, otherwise culturing was performed as described above. ZN positive isolates were identified by AccuProbe *Mycobacterium avium *culture identification test^® ^(GenProbe Inc., San Diego, CA, USA). All isolates were examined for the presence of IS*901 *by PCR using 1 U AmpliTaq^®^DNA polymerase (Applied Biosystems, Foster City, CA, USA). Primers 901a and 901c were used for the amplification of IS*901 *[20]. PCR conditions were as described earlier [18]. The reference strain *M. avium *subsp. *avium *ATCC25291 was used as a positive control, and Milli-Q water as negative control.

**Table 2:** Samples from the Lithuanian pig herd cultured for mycobacteria

Samples	No. examined	No. positive on culture
Lymph node, cervical*	6	6
Lymph node, mesenterial*	5	5
Peat	6	6
Sawdust	4	2
Tap water	4	0
Water pipe	1	0

*Ten pigs were sampled, two isolates originated from different lymph nodes in the same animal

### Restriction Fragment Length Polymorphism (RFLP) on isolates from the Lithuanian herd

IS*1311*- and IS*1245 *RFLP were performed on all isolates as described previously [18,21,22]. The avian reference strain ATTC25291 was used as a positive control and run on each gel [22]. The probes used for IS*1245 *and IS*1311 *RFLP were described by Johansen et al. [18,21]. The resulting RFLP patterns were analysed by visual inspection and by using the BioNumerics software (version 4.0, Applied Maths, Kortrijk, Belgium) as described previously [18]. Optimization and tolerance were set to 1.5% for IS*1311 *RFLP and to 0.8% for IS*1245 *RFLP. With these settings, the reference strain clustered with 100% similarity. A final dendrogram of the composite dataset of the two RFLP experiments was calculated using the similarity by the average from experiments, and the UPGMA clustering method. Correction for internal weights was used. The similarity cut-off was set at 80% to define clusters [18].

## Results

### Tuberculin testing and post mortem examinations of pigs imported to Serbia

Altogether 135 pigs showed positive tuberculin reactions on retesting of the pigs; 130 strong and two weak positive on avian and 77 strong and three weak positive on bovine tuberculin. Seventy-seven animals were positive on both avian and bovine tuberculin, 55 only on avian and three only on bovine. Only 19 of the tested animals showed negative reactions to both avian and bovine tuberculin. Details on reaction patterns against the two types of tuberculin are listed for 92 of the animals in Table 3.

**Table 3:** Results from real time PCR and tuberculin testing of 92 tuberculin positive Serbian pigs

IS*6110 *PCR	IS*1245 *PCR	Bovine tuberculin	Avian tuberculin	No. of animals
+	+	+	+	3
+	+	-	+	4
+	-	+	+	2
+	-	-	+	1
-	+	+	+	22
-	+	-	+	20^a^
-	+	+	-	2
-	-	+	-	1
-	-	+	+	21^b^
-	-	-	+	16^c^

^a^One sample had no pathological lesions

^b^Six samples had no pathological lesions

^c^One sample had no pathological lesions

Post mortem examination was performed on 100 pigs. Granulomatous lesions compatible with mycobacterial infection were found in mesenterial lymph nodes from 88 pigs. Macroscopic lesions were detected from 43 pigs, and only microscopic lesions from 45 pigs. Similar lesions were detected in the ovaries of some sows. In 12 pigs, no lesions were detected. The granulomas were composed of focal accumulation of inflammatory cells in which macrophages, epithelioid cells, multinucleated giant cells and lymphocytes predominated. Caseous necrotic areas were present in some granuloma. Numerous eosinophils and myofibroblasts were present within the granuloma. ZN positive rods were detected in five of the samples. Unfortunately, no growth of mycobacteria was detected in the samples sent for culturing.

### Real-time PCR from FFPE lymph nodes from pigs imported to Serbia

The Norwegian Veterinary Institute received 115 FFPE lymph nodes from the Serbian pigs. Twenty-three samples were excluded from real-time PCR analysis. Only one sample was included from each pig. Additionally, samples with uncertain sample ID or poor DNA quality measured by negative result on real-time PCR for the porcine β-globin gene were excluded. Of the 92 remaining samples analysed for the presence of IS*1245 *by real-time PCR, 51 (55.4%) were regarded positive. Ten (10.9%) of the 92 samples analysed by IS*6110 *real-time PCR were considered positive, all but three were however simultaneously positive in IS*1245 *PCR. The PCR products of the samples positive for IS*6110 *were additionally sequenced and compared to published sequences by NCBI BLAST http://blast.ncbi.nlm.nih.gov/Blast.cgi to confirm their identity. Of the pigs with neither microscopic nor macroscopic lesions, only one of eight samples examined was positive for IS*1245 *by PCR, and none were positive for IS*6110*. In samples from 38 of the tuberculin reactors no IS element could be detected. Responses seen in connection with tuberculin results are given in Table 3.

### Post mortem examinations of animals and examinations of environmental samples from the Lithuanian herd

The lymph nodes collected from clinically healthy pigs in Lithuania were not enlarged and had no external lesions, but on incision, yellowish-white caseous foci of a few millimetres in diameter were revealed on the cut surface of all lymph nodes. *M. avium *was isolated from all lymph nodes examined from the Lithuanian herd, from all samples of peat and from two of the sawdust samples. From the remaining environmental samples, including the water samples, no mycobacteria were detected (Table 2). None of the isolates harboured IS*901*, and they were therefore assigned as *M. avium *subsp. *hominissuis*.

### Restriction Fragment Length Polymorphism (RFLP) analysis of the isolates from the Lithuanian herd

The RFLPs detected between two and eight copies of IS*1311 *and between 11 and 21 copies of IS*1245 *in the isolates. As shown previously [18], the IS*1245 *RFLP showed a higher discriminative power than the IS*1311 *RFLP. Discrimination of the isolates was improved by a concomitant use of IS*1311 *and IS*1245 *RFLP.

**Figure 1:**
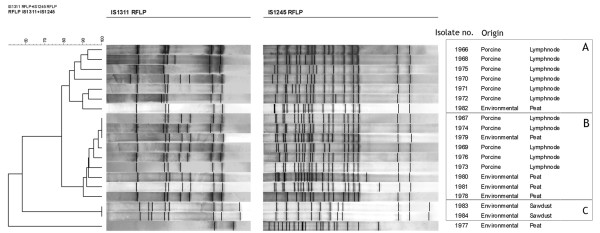
**Cluster analysis based on RFLP analysis of isolates of *Mycobacterium avium *subspecies *hominissuis***. The dendrogram was calculated by using the similarity by the average from experiments and the unweighted-pair group method using arithmetic averages clustering method. Correction for internal weights was used. Two probes were used for RFLP; IS*1311 *and IS*1245*. Identification number and origin of the isolates are listed in columns on the right. Clusters based on 80% similarity are indicated with frames and marked A, B, and C.

Three clusters, where *M. avium *subsp. *hominissuis *isolates shared a similarity of more than 80%, were named A-C (Figure 1). One isolate from peat did not belong to any cluster. Cluster A was composed of six porcine isolates and one from peat, and cluster B consisted of nine isolates; five porcine and four from peat. One isolate from peat (#1979) was identical to two porcine isolates (#1967 and # 1974). The two isolates originating from sawdust belonged to cluster C. They showed identical RFLP profiles that were different from the porcine isolates. Isolate #1966 and #1976 originated from the same pig, but clustered in different clusters.

Eight isolates had identical profiles by IS*1311 *RFLP; four porcine (#1967, #1969, #1974 and #1976) and four peat isolates (# 1978, #1979, #1980 and #1981). In the IS*1245 *RFLP, however, only three of these isolates showed identical profiles (#1967, #1974 and #1979). The other five isolates showed differences of one to three bands compared to the profile of the three identical isolates (Figure 1).

## Discussion

Tuberculin reactions and pathological lesions in the pigs imported to Serbia indicated mycobacterial infection. More than 50% of the animals reacted positive for both avian and bovine tuberculin. Only three pigs reacted positive to bovine and negative to avian tuberculin. None of the FFPE lymph nodes originating from these three pigs were positive on real time PCR for IS*6110*, while two were positive for IS*1245*. Cross reactions between avian and bovine tuberculin are commonly observed in cattle [10], and our results indicate that such reactions are common in pigs as well.

In all but 12 pigs, pathological lesions were detected, indicating a very high infection rate in the herd. Only one of the PCR examined FFPE lymph nodes from the pigs without pathological lesions was positive for IS*1245 *by PCR, confirming that a thorough macroscopic and microscopic examination will detect most of the infected pigs. However, neither tuberculin reactions nor pathology can differentiate between infections with *M. bovis *and *M. avium*.

Culturing was unsuccessful, possibly due to low number of bacteria in the samples, uneven distribution of bacteria in the lesions or the lack of different growth media used for culture. As the samples were discarded, reculturing was not possible. Still, positive reactions for IS*1245 *on real-time PCR confirmed presence of *M. avium *in 55% of the FFPE lymph nodes. It was therefore assumed that the animals imported to Serbia were infected with *M. avium*.

Real-time PCR for IS*6110 *gave positive reactions in FFPE lymph nodes from ten pigs in Serbia. This might indicate an infection with the MTC, possibly before import. However, this is unlikely as Lithuania is officially free of bovine tuberculosis today and the last occurrence in domestic animals was reported in 2001 [23,24]. In Serbia, bovine tuberculosis is still present in domestic animals [23], but considering the relatively short lapse of time between import to Serbia and sampling of the lymph nodes, and the strict quarantine measures applied, it seems unlikely that the animals were infected with MTC after import. The results are possibly due to false positive reactions or to cross reactions with DNA sequences yet to be described. However, without the possibility to culture mycobacteria from the IS*6110 *positive lymph nodes, it is not possible to conclude with 100% certainty whether any of these pigs were infected by bacteria within the MTC.

The sensitivity and specificity of the real-time PCRs for IS*6110 *and IS*1245 *cannot be determined as culturing in Serbia was not successful for these samples. Real-time PCR is considered an efficient, highly sensitive and specific diagnostic tool, using the appropriate controls and protocols. However, false negatives on real-time PCR might be due to impaired DNA quality, as formalin fixation and paraffin embedding are reported to compromise the integrity of DNA [25]. To counteract the negative effect of formalin fixation and paraffin embedding on DNA quality, the target sequences were designed to be short (≤ 200 bp) and samples with Ct values up to 40 were considered positive. These measures lower the possibility of false negatives, but also increase the risk of false positive results. Seven of the ten samples positive for IS*6110 *were also positive for IS*1245*. This could indicate multiple infections or false positive reactions. The specificity of IS*6110 *PCR in clinical material have been described to be between 96.1% and 100% [26,27], but could be lower for real time PCR in FFPE lymph nodes.

The animals were kept in quarantine in Serbia, and the first tuberculin testing was performed only 10 days after arrival. It was therefore assumed that any mycobacterial infection was contracted in Lithuania before export. Through culture and RFLP analysis of samples from the Lithuanian breeding facility, this suspicion was further confirmed, as *M. avium *subsp. *hominissuis *was detected in several samples from pigs and from their environment. The lymph nodes sampled from the Lithuanian breeding herd were randomly collected from healthy swine at slaughter, showing no visible external lesions. Still, all were positive for *M. avium *subsp. *hominissuis*, indicating a very high level of infection in the herd. *M. avium *subsp. *hominissuis *was also isolated from half of the sawdust and all peat samples, showing that the pigs lived in a contaminated environment.

Identical and closely related isolates from peat and porcine lymph nodes were detected by RFLP analysis, indicating peat as a possible source of infection for the pigs. Four porcine and four peat isolates identical on IS*1311 *RFLP, showed minor differences in the IS*1245 *RFLP profiles. Such differences have earlier been observed both on subculture and in multiple cultures from the same individual [28,29]. Tenover et al. [30] demonstrated how one mutation could result in two or three band differences in pulsed field gel electrophoresis patterns and that such patterns could be considered subtypes of the outbreak pattern when isolates are epidemiologically linked. A similar interpretation may be valid for RFLP patterns, which would indicate a close genetic relationship between these eight isolates. The discrepancies in RFLP profiles between porcine isolates and isolates originating from sawdust indicate that mycobacteria in sawdust were not the source of infection in the pigs. Two isolates originating from the same animal clustered in two different clusters. This suggests an infection with two different variants, and could be related to a high infection pressure in the environment. Such infections have been described earlier, both in humans and in swine [31-34].

In the Lithuanian herd, iron enriched peat was fed to piglets as an iron supplement and for regulation of intestinal function, while sawdust was used for bedding. *M. avium *subsp. *hominissuis *has been isolated from both peat and sawdust used in swine farms earlier, and related RFLP types have been detected in both peat and swine and in sawdust and swine [35-37]. Feeding contaminated peat to small piglets could explain the high level of infection in this herd, because young animals are more susceptible to infectious agents than older animals. After the source of infection was identified, all peat and sawdust was removed, the facilities were cleaned and disinfected, and new heat treated peat was introduced in the herd.

## Conclusions

Cross reaction between avian and bovine tuberculin was demonstrated in pigs imported from Lithuania to Serbia. Real time PCR analysis of FFPE lymph nodes from pigs positive on both tuberculins indicated infection with *M. avium*. However, a small proportion of FFPE samples were PCR positive for MTC. *M. avium *subsp. *hominissuis *was subsequently detected by culture from randomly selected porcine lymph nodes and from peat and sawdust from the Lithuanian herd facilities. Isolates from pigs and peat showed genetic relationship by RFLP, suggesting that the infection probably originated from contaminated peat and that the exported pigs were infected by *M. avium *subsp. *hominissuis *at an early age.

## Authors' contributions

AA and TBJ contributed to conception and design, culture and molecular studies, data analyses and drafting of the manuscript. VP, SAK and DV contributed to tuberculin testing, pathological and histopathological analyses and writing of manuscript. AJ and JŽ contributed to collection of samples and writing of manuscript. SFN contributed to molecular studies and writing of manuscript. BL contributed to conception and design, pathological analyses and writing of the manuscript. GH and BKD contributed to conception and design, analysed data and helped to draft the manuscript. All authors have read and approved the final manuscript.
